# Assembling the Pieces of a Systematic Review: A Guide for Librarians

**DOI:** 10.5195/jmla.2018.462

**Published:** 2018-07-01

**Authors:** Gerald Natal

**Affiliations:** Mulford Health Science Library, University of Toledo, Toledo, OH

In the preface to the book *Assembling the Pieces of a Systematic Review: A Guide for Librarians,* the editors quote Ben Goldacre (p. xiii), who maintains that the systematic review is “one of the most important innovations in medicine” in recent history. Given the importance of systematic reviews for evidence-based decision-making, education on sound execution is essential. While books exist that address the systematic review for specific disciplines (nursing, students, social sciences, health sciences), this book places particular emphasis on roles for librarians. However, any reader who is interested in learning how to complete a successful review should find this book insightful.

Chapters are arranged thoughtfully, beginning with definitions and background information, followed by three main sections concerning planning, identifying, and evaluating reviews. These chapters cover aspects of conducting reviews that researchers often overlook, such as the appropriateness of the research question, the importance of reducing bias, and the use of grey literature. A chapter is dedicated to planning a systematic review service, with details of various models and marketing strategies. The final chapter discusses impediments to the systematic review process and development approaches. The book concludes with an examination of systematic reviews in librarianship.

Each chapter begins with a relatable quote and contains objectives, an overview, and references. Readers should find the chapter with the variety of case studies especially useful. Each case provides a valuable example of a review strategy, a format for framing the research question, and a list of available related resources and readings. The book is replete with checklists, recommendations for useful tools, lists of organizations that supply evidence-based information and guidelines for reporting, and resources for staying current with standards. Throughout the book are “action boxes” to stimulate critical evaluation of reviews, and a “Librarian’s Corner” to draw attention to information of particular interest to librarians. The authors attempt to cover every aspect of the systematic review process, including addressing the vexing question “when to stop searching” the literature.

*Assembling the Pieces of a Systematic Review: A Guide for Librarians* is a well-written book by qualified authors that serves as a manual for conducting reviews or forming and managing a review service, regardless of skill level. Readers should gain an appreciation of the scope of systematic reviews and a better understanding of contributions that librarians can make that go beyond those typically made.

